# The clinical outcomes of laparoscopic proximal gastrectomy with double-tract reconstruction versus tube-like stomach reconstruction in patients with adenocarcinoma of the esophagogastric junction based on propensity score-matching: a multicenter cohort study

**DOI:** 10.3389/fonc.2023.1137836

**Published:** 2023-06-02

**Authors:** Zhiwen Xu, Jinping Chen, Shaoqin Chen, Hexin Lin, Kang Zhao, Changyue Zheng, Huibin Liu, Zhihua Chen, Yongan Fu, Qingqi Hong, Wei Lin, Su Yan, Jun You

**Affiliations:** ^1^ Department of Gastrointestinal Oncology Surgery, the First Affiliated Hospital of Xiamen University, Xiamen, China; ^2^ School of Medicine, Xiamen University, Xiamen, China; ^3^ Department of Gastrointestinal Surgery, the First Hospital of Quanzhou, Quanzhou, China; ^4^ Department of Gastrointestinal Surgery, the First Affiliated Hospital of Fujian Medical University, Fuzhou, China; ^5^ Department of Gastrointestinal Oncology Surgery, the Affiliated Hospital of Qinghai University, Xining, China; ^6^ Department of Gastrointestinal Surgery, the Affiliated Hospital of Putian College, Putian, China

**Keywords:** adenocarcinoma of the esophagogastric junction, proximal gastrectomy, digestive tract reconstruction, double-tract reconstruction, tube-like stomach reconstruction, quality of life

## Abstract

**Purpose:**

Laparoscopic proximal gastrectomy with double-tract reconstruction (LPG-DTR) and laparoscopic proximal gastrectomy with tube-like stomach reconstruction (LPG-TLR) are both function-preserving procedures performed for treating AEG. However, there is no clinical consensus on the selection of digestive tract reconstruction after proximal gastrectomy, and the best way to reconstruct the digestive tract remains controversial. This study aimed at comparing the clinical outcomes of LPG-DTR and LPG-TLR to provide some reference to the choice of AEG surgical modalities.

**Methods:**

This was a multicenter, retrospective cohort study. we collected clinicopathological and follow-up data of patients with consecutive cases diagnosed with AEG from January 2016 to June 2021 in five medical centers. According to the way of digestive tract reconstruction after tumor resection, patients who underwent LPG-DTR or LPG-TLR were included in the present study. Propensity score matching (PSM) was performed to balance baseline variables that might affect the study outcomes. The QOL of the patients was evaluated using the Visick grade.

**Results:**

A total of 124 eligible consecutive cases were finally included. Patients in both groups were matched using the PSM method, and 55 patients from each group were included in the analysis after PSM. There was no statistically significant difference between the two groups in terms of the operation time, amount of intraoperative blood loss, days of postoperative abdominal drainage tube placement, postoperative hospitalization days, total hospitalization cost, the total number of lymph nodes cleared, and the number of positive lymph nodes (*P*>0.05). There was a statistically significant difference between the two groups in terms of time to first flatus after surgery and postoperative soft food recovery time (*P*<0.05). For the nutritional status, the weight levels at 1 year after surgery was better in the LPG-DTR group than in the LPG-TLR group (*P*<0.05). There was no significant difference in Visick grade between the two groups (*P*>0.05).

**Conclusion:**

The anti-reflux effect and quality of life of LPG-DTR for AEG were comparable to those of LPG-TLR. Compared with LPG-TLR, LPG-DTR provide better nutrition status for patients with AEG. LPG-DTR is a superior reconstruction method after proximal gastrectomy.

## Introduction

Adenocarcinoma of the esophagogastric junction (AEG) has become more common in recent years (1, 2), and data from studies in several countries worldwide show that its incidence is increasing, posing a significant threat to human health (3–5). The clinical treatment for AEG is different from that for gastric cancer due to the distinctions in tumor pathology and the location of the disease (6, 7). At present, there is no uniform consensus on the standardized treatment of AEG diseases, and many issues are controversial.

Surgery is still an important method for the treatment of AEG, and the focus of attention in the surgical field is mainly on the extent of resection and the choice of digestive tract reconstruction after tumor resection. After proximal gastrectomy, digestive tract reconstruction is mainly divided into two categories: esophageal-gastric anastomosis and esophageal-jejunal anastomosis, with the former mainly represented by Kamikawa, Tube-like stomach, and Side overlap reconstruction, and the latter mainly represented by double-tract and interposition jejunal reconstruction (8–10).

Different methods of digestive tract reconstruction after proximal gastrectomy have their advantages and disadvantages. However, prospective, large-sample, and multicenter studies are still lacking. There is no consensus on the best method for digestive tract reconstruction after proximal gastrectomy.

In this study, we combined the clinicopathological and follow-up data of AEG patients admitted to five medical centers in China in recent years, and retrospectively analyzed two types of reconstruction methods, laparoscopic proximal gastrectomy with double-tract reconstruction (LPG-DTR) and laparoscopic proximal gastrectomy with tube-like stomach reconstruction (LPG-TLR), which are currently relatively promising in current clinical practice, using the PSM method. Hope to be able to provide some reference to the choice of AEG surgical modalities.

## Materials and methods

### Study design

This was a multicenter, retrospective cohort study. In this study, we collected clinicopathological and follow-up data of patients with consecutive cases diagnosed with AEG from January 2016 to June 2021 in five medical centers in China. Patients who underwent LPG-DTR or LPG-TLR were included in the present study. The participating institutions are high-volume cancer surgery centers. Propensity score matching (PSM) was performed to minimize the differences between the LPG-DTR and LPG-TLR groups (11). The 12 variables used to calculate the propensity score included gender, age, height, weight, BMI, preoperative glucose, preoperative hemoglobin, preoperative total protein, preoperative albumin, tumor length diameter, histological differentiation, and pathological TNM stage. The study was approved by the Institutional Review Board at the First Affiliated Hospital of Xiamen University.

### Patient population

The eligibility criteria were patients 1) diagnosed with AEG Siewert II, Siewert III type by tissue biopsy; 2) who underwent radical proximal gastrectomy; and 3) with no distant metastases detected by preoperative CT, MRI, and other imaging examinations.

The exclusion criteria included patients 1) with laparotomy surgery; 2) with a history of malignancy or other organs complicated with malignant tumor; 3) with a history of gastrectomy; 4) who required combined resection due to other diseases; and 5) who had a complete absence of clinicopathological and follow-up data.

### Surgical procedure

All patients underwent laparoscopic radical proximal gastrectomy by physicians experienced in gastrointestinal surgery at each center. We required that the attending surgeons have more than 100 cases of laparoscopic radical gastrectomy for gastric cancer. The tissues and organs were freed and D1+ lymph nodes were cleared under laparoscopy. When the tumor invades the esophagus at a distance greater than or equal to 4 cm, upper, middle, and lower mediastinal lymph node dissection is performed simultaneously, and when the tumor invades the esophagus at a distance greater than or equal to 2 cm, lower mediastinal lymph node dissection is performed.

LPG-DTR: After deflating the pneumoperitoneum, the proximal stomach with the tumor was resected, and the jejunum was cut at 20 cm from the flexural ligament. The distal jejunum was performed with the esophagus by end-lateral anastomosis. The side of the large curvature of the residual stomach was performed with the jejunum at 10 to 15 cm from the esophagojejunostomy by lateral anastomosis. The proximal jejunum was anastomosed with the small intestine about 30 to 35 cm away from the gastrointestinal anastomosis. And all anastomoses and closures were reinforced with sutures.

LPG-TLR: After deflating the pneumoperitoneum, an 8 to 10 cm long adjuvant incision was made in the epigastrium to remove the proximal stomach with tumor, and a linear cutting closure was used to cut the remnant stomach along the less curved side at 3 to 5 cm above the pylorus to make a tubular shape about 20 cm long and 4 cm wide, and a circular anastomosis was used to anastomose the esophagus and the anterior end of the tubular stomach. All surgical operations followed the basic principles of the *Esophageal and Esophagogastric Junction Cancers* and the *Japanese Gastric Cancer Treatment Guidelines 2018* (*5th edition*) (12, 13).

### Endpoints

This study’s primary endpoints were comparing changes in Nutrition-related indicators and the QoL of patients between the LPG-DTR and LPG-TLR groups. The secondary endpoints were the postoperative complications, frequency of reflux esophagitis, and perioperative outcomes.

Short-term outcomes: operation time, intraoperative bleeding, time to first flatus after surgery, duration of postoperative abdominal drainage, postoperative soft food recovery time, the number of postoperative hospital days, total number of lymph nodes obtained, number of positive lymph nodes, and perioperative complications. Postoperative complications were classified by Clavien-Dindo (CD) classification (14). Short-term complications were defined as those that occurred<30 days after surgery, and those occurring subsequently were defined as Long-term complications.

Long-term condition: postoperative changes in weight, hemoglobin, and albumin, postoperative gastroesophageal reflux, anastomotic stricture, and other long-term complications. Since some patients did not undergo postoperative gastroscopic examination, gastroesophageal reflux was assessed by a combination of gastroscopy and the GERDQ scale to avoid bias (15). The QOL of the patients was evaluated using the Visick grade (16, 17). The Visick grade was divided into four grades: Visick I refers to good postoperative recovery without significant discomfort; Visick II refers to occasional symptoms such as bloating and diarrhea that do not interfere with daily life and work; Visick III refers to mild to moderate dumping syndrome, reflux esophagitis and other symptoms that require medication but allow normal life and work; Visick IV refers to moderate to severe symptoms or significant complications that interfere with normal life and work.

Postoperative nutritional indicators were obtained from outpatient or inpatient examination medical records at 6 months and 1 year postoperatively.

The discharge criteria included the following (1): Patients can eat food normally through the mouth (2); The abdominal drainage tube and all other drainage devices were removed (3); All perioperative complications were cured (4); The patient has no obvious symptoms of discomfort.

### Follow-up

Follow-up was performed for all patients after discharge from the hospital via telephone, outpatient visits, and inpatient examination. It was conducted every 3 months for the first 2 years postoperatively and every 6 months thereafter. And they were followed regularly with the same protocol. The follow-up period was up to July 1, 2022, and the results of the one-year postoperative follow-up were used for the Visick grade assessment.

### Statistical analysis

The SPSS 26.0 statistical software was used to perform all statistical analyses, and propensity score matching was performed 1:1 by the nearest neighbor matching method with a caliper value set at 0.2. Normally distributed measures were expressed as the means ± standard deviations, and a t-test was used for comparison between groups. The measures of skewed distribution were expressed as M(range), and the nonparametric test was used for comparison between groups. Categorical data data were expressed as frequencies and percentages, and comparisons between groups were made using the Chi-square, Fisher’s exact probability method, or Mann-Whitney U test. All values were double-tailed, and P values<0.05 were considered significant.

## Results

### Baseline information before and after propensity score matching

After removing unqualified cases according to the inclusion and exclusion criteria, a total of 124 eligible consecutive cases were finally included, all of whom underwent LPG-DTR or LPG-TLR, including 51 cases in the First Affiliated Hospital of Xiamen University, 49 cases in the Affiliated Hospital of Qinghai University, 15 cases in the Affiliated Hospital of Putian College, 5 cases in the First Hospital of Quanzhou, and 4 cases in the First Affiliated Hospital of Fujian Medical University. Of the 124 patients eventually enrolled, 55 patients underwent LPG-DTR, and 69 patients underwent LPG-TLR. Patients in both groups were matched using the PSM method. Finally, 55 patients from each group were included in the analysis after PSM.

**Table 1** shows prematching baseline characteristics of the overall study population of 124 patients. All 124 patients underwent curative (R0) surgery for AEG, and no patient died in the perioperative period. **Table 2** shows postmatching baseline characteristics of 55 patients undergoing LPG-DTR and 55 patients undergoing LPG-TLR. After matching, there were no significant differences in the baseline characteristics between the two groups (*P*>0.05), which were well-balanced.

**Table 1:** Basic characteristics of the patients before propensity score matching.

	LPG-DTR (n=55)	LPG-TLR (n=69)	P value
Age (years)	62.2 ± 8.9	62.3 ± 9.848	0.975
Sex			0.953
Female	13	16	
Male	42	53	
Height (cm)	166.9 ± 7.8	166.2 ± 6.6	0.650
Weight (kg)	63.8 ± 11.2	62.2 ± 9.8	0.396
Preoperative BMI (kg/m^2^)	23.0 ± 4.0	22.5 ± 3.2	0.447
Glucose (μmol/L)	5.3 ± 1.1	5.3 ± 1.7	0.806
Hemoglobin (g/L)	136.6 ± 19.4	138.2 ± 24.6	0.682
Total protein (g/L)	69.9 ± 8.0	68.7 ± 7.2	0.419
Albumin (g/L)	41.7 ± 4.5	40.6 ± 4.1	0.148
Tumor size (cm)	2.4 ± 1.0	3.2 ± 1.9	0.004
pTNM stage			
I	40	33	0.006
II	9	21	
III	6	15	
Histology			
well differentiated	18	11	0.037
moderately differentiated	28	41	
poorly/undifferentiated differentiated	9	17	

Data are shown as mean ± SD or number (%).

LPG-DTR, laparoscopic proximal gastrectomy with double-tract reconstruction.

LPG-TLR, laparoscopic proximal gastrectomy with tube-like stomach reconstruction.

BMI, body mass index; TNM, tumor-node-metastasis. TNM staging was performed according to the AJCC 7th edition.

**Table 2:** Basic characteristics of the patients after propensity score matching.

	LPG-DTR (n=55)	LPG-TLR (n=55)	P value
Age (years)	62.2 ± 8.9	61.9 ± 9.3	0.859
Sex			0.820
Female	13	12	
Male	42	43	
Height (cm)	166.8 ± 7.8	166.2 ± 6.5	0.644
Weight (kg)	63.8 ± 11.2	62.1 ± 9.6	0.400
Preoperative BMI (kg/m^2^)	23.0 ± 4.0	22.5 ± 3.0	0.453
Glucose (μmol/L)	5.3 ± 1.1	5.3 ± 1.6	0.864
Hemoglobin (g/L)	136.6 ± 19.4	137.5 ± 23.4	0.821
Total protein (g/L)	69.9 ± 8.0	68.6 ± 7.0	0.365
Albumin (g/L)	41.6 ± 4.5	40.9 ± 4.4	0.381
Tumor size (cm)	2.4 ± 1.0	2.8 ± 1.7	0.222
pTNM stage			
I	40	30	0.061
II	9	16	
III	6	9	
Histology			
well differentiated	18	10	0.139
moderately differentiated	28	34	
undifferentiated/poorly differentiated	9	11	

Data are shown as mean ± SD or number (%).

LPG-DTR, laparoscopic proximal gastrectomy with double-tract reconstruction.

LPG-TLR, laparoscopic proximal gastrectomy with tube-like stomach reconstruction.

BMI, body mass index; TNM, tumor-node-metastasis. TNM staging was performed according to the AJCC 7th edition.

### Intraoperative and postoperative conditions

The perioperative results are listed in **Table 3**. There were no statistical differences between the two groups in terms of the operation time, amount of intraoperative blood loss, days of postoperative abdominal drainage tube placement, postoperative hospitalization days, total hospitalization cost, the total number of lymph nodes cleared, and the number of positive lymph nodes (*P*>0.05). There was a statistically significant difference between the two groups in terms of time to first flatus after surgery and postoperative soft food recovery time (*P*<0.05). The time to first flatus after surgery and time to first soft diet postoperative were significantly lower in the LPG-DTR group than in the LPG-TLR group.

**Table 3:** Surgical outcomes after propensity score matching.

	LPG-DTR (n=55)	LPG-TLR (n=55)	P value
Operation time (min)	262.6 ± 61.2	261.9 ± 42.4	0.941
Intraoperative blood loss (ml)	102.8 ± 122.0	126.0 ± 91.5	0.262
Time to first flatus after surgery (days)	3.6 ± 2.2	5.4 ± 2.4	0.000
Time to first soft diet (days)	7.4 ± 3.8	9.0 ± 3.0	0.014
Removal of abdominal drainage (days)	9.1 ± 7.1	8.8 ± 3.3	0.796
Postoperative hospital stay (days)	15.6 ± 9.3	18.5 ± 7.8	0.080
Number of retrieved lymph nodes	21.1 ± 9.7	24.3 ± 9.7	0.094
Number of positive lymph nodes	0.5 ± 1.5	1.2 ± 2.7	0.074

Data are shown as mean ± SD.

LPG-DTR, laparoscopic proximal gastrectomy with double-tract reconstruction.

LPG-TLR, laparoscopic proximal gastrectomy with tube-like stomach reconstruction.

### Postoperative complications

Postoperative complications are shown in **Table 4**. Among the short-term complications, 7 cases of pulmonary infection, 6 cases of pleural effusion, 2 cases of wound infection, 1 case of intra-abdominal bleeding, and 3 cases of anastomotic leakage occurred in the LPG-DTR group, and the above complications were 7, 4, 4, 4 and 4 cases in the LPG-TLR group, respectively. When compared by Clavien-Dindo classification, there were 2 cases of grade I, 9 cases of grade II, and 4 cases of grade III in the LPG-DTR group and 2 cases of grade I, 2 cases of grade II, and 9 cases of grade III in the LPG-TLR group, and there were no patients with grade IV and V in both groups. The overall incidence of short-term complications was 27.3% in the LPG-DTR group and 23.6% in the LPG-TLR group, and we found no significant differences between the two groups in the Clavien-Dindo classification of short-term complications (*P*= 0.944).

**Table 4:** Postoperative complications after propensity score matching.

	LPG-DTR (n=55)	LPG-TLR (n=55)	P value	
Short-term postoperative complications				
No	40 (72.7)	42 (76.4)		
Yes	15 (27.3)	13 (23.6)		
Pulmonary infection	7 (12.7)	7 (12.7)	1.000	
Pleural effusion	6 (10.9)	4 (7.3)	0.507	
Wound infection	2 (3.6)	4 (7.3)	0.679	
Anastomotic leakage	3 (5.5)	4 (7.3)	1.000	
Intra-abdominal bleeding	1 (1.8)	4 (7.3)	0.363	
Clavien–Dindo classifification				
Grade 1	2 (3.6)	2 (3.6)	0.082	
Grade 2	9 (16.4)	2 (3.6)		
Grade 3	4 (7.3)	9 (16.4)		
Grade 4	0	0		
Long-term postoperative complications				
No	46 (83.6)	39 (70.9)	
Yes	9 (16.4)	16 (29.1)	
Reflux esophagitis	6 (10.9)	10 (18.2)	0.279	
Anastomotic stenosis	3 (5.5)	5 (9.1)	0.716	
Intestinal obstruction	2 (3.6)	3 (5.5)	1.000	

Data are shown as number (%).

LPG-DTR, laparoscopic proximal gastrectomy with double-tract reconstruction.

LPG-TLR, laparoscopic proximal gastrectomy with tube-like stomach reconstruction.

There were 4 persons with CD grade III in the LPG-DTR group, including 2 persons with anastomotic leakage, 1 with intra-abdominal bleeding, and 1 with anastomotic leakage combined with pleural effusion. There were 9 persons with CD grade III in the LPG-TLR group, including 2 persons with pleural effusion, 3 persons with anastomotic leakage, 3 persons with intra-abdominal bleeding, and 1 with anastomotic leakage combined with intra-abdominal bleeding. In the above patients, the treatment for patients who developed pleural effusion was thoracic drainage. Patients who developed anastomotic leakage were treated with abdominal irrigation and drainage. After the abdominal bleeding was diagnosed, the patient was admitted to the operating room for laparoscopic exploration to stop the bleeding. All of the above complications were cured with surgical intervention.

Among the long-term complications, reflux esophagitis occurred in 6 (10.9%) cases in the LPG-DTR group and 10 (18.2%) cases in the LPG-TLR group, respectively, and there was no statistically significant difference between the two groups (*P*=0.279). Three (5.5%) cases of anastomotic stenosis occurred in the LPG-DTR group, and 5 (9.1%) cases of anastomotic stenosis occurred in the LPG-TLR group, and there was no significant difference between the two groups (*P*=0.716). Postoperative intestinal obstruction occurred in 2 cases in the LPG-DTR group and 3 cases in the LPG-TLR group, and there was no significant difference between the two groups (*P*=1.000).

### Nutrition-related indicators

**Figure 1:**
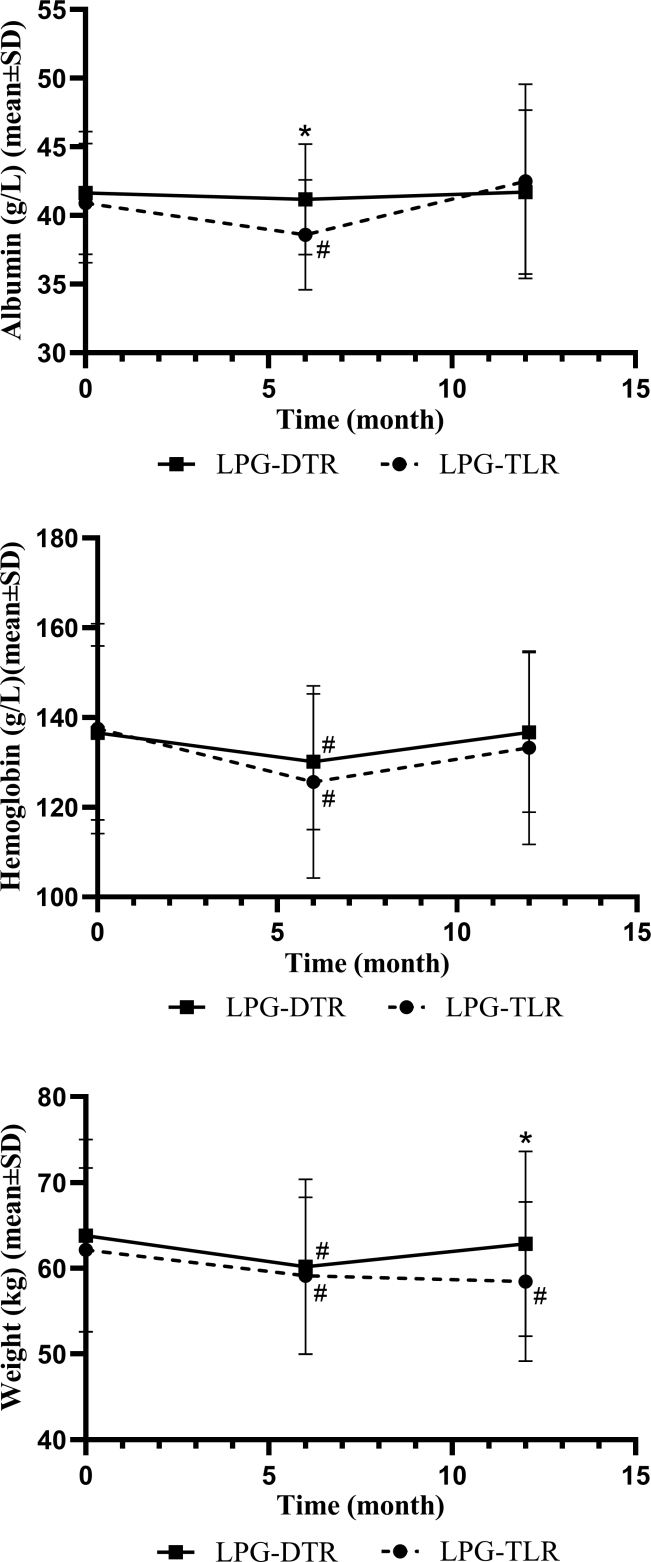
Nutritional indicators of LPG-DTR and LPG-TLR after propensity score matching (weight, hemoglobin, albumin). LPG-DTR: laparoscopic proximal gastrectomy with double-tract reconstruction. LPG-TLR, laparoscopic proximal gastrectomy with tube-like stomach reconstruction. *P less than 0.05 between two groups, #P less than 0.05 comparison with preoperative.

Nutritional indicators for both groups are shown in **Table 5** and **Figure 1**. The weight, hemoglobin, and albumin levels of the patients at 6 months and 1 year postoperative were counted, respectively. In the condition of no significant difference in preoperative parameters, the results of the comparison between the two groups showed that there were no significant differences in hemoglobin and albumin levels between the two groups 1 year after surgery (*P*>0.05). However, there was a significant difference in indices related to weight between the two groups 1 year after surgery, with patients in the LPG-DTR group having higher weight levels 1 year after surgery as compared to that of their counterparts in the LPG-TLR group.

**Table 5:** Nutritional indicators after propensity score matching.

		LPG-DTR (n=55)	LPG-TLR (n=55)	P value
Weight (kg)	Preoperative	63.8 ± 11.2	62.1 ± 9.6	0.400
	6 months after the operation	60.2 ± 10.2	59.1 ± 9.1	0.597
	1 year after the operation	62.8 ± 10.8	58.4 ± 9.3	0.030
Hemoglobin (g/L)	Preoperative	136.6 ± 19.4	137.5 ± 23.4	0.821
	6 months after the operation	130.2 ± 15.1	125.6 ± 21.4	0.270
	1 year after the operation	136.7 ± 17.8	133.3 ± 21.5	0.528
Albumin (g/L)	Preoperative	41.6 ± 4.5	40.9 ± 4.4	0.381
	6 months after the operation	41.8 ± 5.3	39.9 ± 4.9	0.005
	1 year after the operation	42.2 ± 7.6	44.2 ± 9.1	0.666

Data are shown as mean ± SD.

LPG-DTR, laparoscopic proximal gastrectomy with double-tract reconstruction.

LPG-TLR, laparoscopic proximal gastrectomy with tube-like stomach reconstruction.

We further compared these nutritional parameters with the preoperative period in each group. Only patients in the LPG-TLR group had a significant weight loss 1 year after surgery compared to preoperative levels, whereas the LPG-DTR group was able to return to preoperative levels. In the patients of both groups, hemoglobin and albumin levels could be recovered to preoperative levels 1 year after surgery.

### Postoperative quality of life

The postoperative QOL was assessed by Visick grade. These outcomes are listed in **Table 6**. In the QoL analysis, the percentage of Visick I, II, III, and IV in the LPG-DTR group was 50.9% (28/55), 34.5% (19/55), 14.5% (8/55), and 0% (0/55), respectively, while those of grade for the LPG-TLR group was 56.4% (31/55), 23.6% (13/55), 20.0% (11/55), and 0% (0/55), respectively. There was no significant difference in the overall Visick grade between the two groups (*P*>0.05). Further, the proportion of those with Visick III-IV was no significant difference between the two groups as well (*P*>0.05), although the percentage of the above indicators was 14.5% and 20.0% in the two groups, respectively. Patients in both groups were predominantly Visick I-II, and they were asymptomatic or mildly symptomatic patients who did not require additional intervention in their daily lives.

**Table 6:** The Visick grade of the two groups after propensity score matching.

	LPG-DTR (n=55)	LPG-TLR (n=55)	P value
I	28 (50.9%)	31 (56.4%)	0.843
II	19 (34.5%)	13 (23.6%)	
III	8 (14.5%)	11 (20.0%)	
IV	0 (0%)	0 (0%)	
≥III	8 (14.5%)	11 (20.0%)	0.449

Data are shown as number (%).

LPG-DTR = laparoscopic proximal gastrectomy with double-tract reconstruction.

LPG-TLR = laparoscopic proximal gastrectomy with tube-like stomach reconstruction.

## Discussion

Previous studies have considered total gastrectomy as one of the standard treatment modalities for gastric cancer, and it is also widely used in AEG. In recent years, some studies have concluded that preserving part of the stomach can lead to a better postoperative nutritional status of patients (18). In the 6th edition of the Japan Gastric Cancer Treatment Guidelines, updated in 2021 (19), the recommended standard surgical procedure for upper gastric cancer is total gastrectomy, while proximal gastrectomy is also recommended in two situations. In the first case, proximal gastrectomy is feasible for patients with cT1N0 staging, where more than 50% of the stomach can be preserved after surgery; in the second case, proximal gastrectomy is feasible for patients with cT2+ or N+ staging if the tumor diameter is less than 4 cm, but there is no consensus on the specific way to reconstruct the digestive tract after tumor resection.

Some studies have shown that laparoscopic proximal gastrectomy is as safe and feasible as total gastrectomy, and survival rates of patients with proximal gastrectomy are comparable to that of patients with total gastrectomy (20–23).

The frequent reflux symptoms and resulting poorer QoL of the esophagogastrostomy of the digestive tract are now recognized by most surgeons, and it is now less commonly performed in clinical treatment. As early as 2014, Korean surgeons retrospectively reported 43 cases of double-tract reconstruction after proximal gastrectomy, and their study concluded that double-tract reconstruction is a simple and accessible surgical approach with good anti-reflux results (24). In recent years, double-tract reconstruction of the digestive tract after proximal gastrectomy has been gradually carried out in some treatment centers, and several retrospective studies have shown that its anti-reflux effect is better than that of traditional esophagogastrostomy reconstruction, and it is a relatively prospective reconstruction method with wide clinical application among various digestive tract reconstruction methods (25–28).

Meanwhile, LPG-TLR, in which the stomach is made into a tubular shape and then anastomosed to the esophagus, is preferred in some clinical centers because of its ease of operation and acceptable anti-reflux effect (29). In this study, we selected LPG-DTR and LPG-TLR to compare differences between the two digestive tract reconstruction methods in terms of perioperative recovery, postoperative anti-reflux effect, nutrition-related index changes, and QoL. We are to provide some references for the treatment of AEG and the choice of surgical procedure.

### Perioperative clinical outcomes

In terms of perioperative clinical outcomes, the results of our study showed no significant differences between LPG-DTR and LPG-TLR in terms of the operation time, amount of intraoperative blood loss, days of postoperative abdominal drainage tube placement, and postoperative hospitalization days indicating that the two reconstructive approaches have similar outcomes in most of the perioperative indicators.

Our results showed no significant difference in the incidence of anastomotic leakage between the two reconstructive methods, with an incidence of 5.5% and 7.3%, respectively. More careful intraoperative anatomical separation and protection of tissues, vessels, and nerves, and reinforcement of the anastomotic suture, all of which can effectively reduce the probability of postoperative anastomotic complications and improve the safety of the surgery. In our study, the overall perioperative complication rate was 27.3% in the LPG-DTR group and 23.6% in the LPG-TLR group, with no significant difference between the two groups.

In the KLASS05 multicenter randomized controlled trial (30), the perioperative complication rate was 23.5% in the LP-DTR group, which included 63 patients. This is similar to our results. We believe that the complexity of the procedure does not necessarily increase the risk of postoperative complications, and that careful intraoperative manipulation can effectively reduce the occurrence of operation-related complications. In a controlled study of double-tract reconstruction versus esophagogastrostomy reconstruction, the relevant results corroborated our view (31).

### Gastrointestinal reflux

Gastroesophageal reflux is an important factor affecting the postoperative QoL in patients undergoing proximal gastrectomy, and the results of our study showed that the incidence of reflux esophagitis was 10.9% in the LPG-DTR group and 18.2% in the LPG-TLR group. Although the incidence in the LPG-TLR group was slightly higher than that in the LPG-DTR group, the difference between the two groups still did not reach a statistical difference, and the incidence of reflux esophagitis in both reconstruction modalities was much lower than that of 32-74% in conventional esophagogastrostomy reconstruction (32, 33). These results suggest that the anti-reflux effect of both LPG-DTR and LPG-TLR is superior to that of esophagogastrostomy reconstruction.

LPG-DTR reduces the incidence of reflux esophagitis by placing a 10 to 15 cm long section of jejunum between the remnant stomach and esophagus, thus achieving a better anti-reflux effect. One study showed that the incidence of reflux esophagitis was only 11.7% in the 1st year after double-tract reconstruction (34). In another controlled study of double-tract reconstruction versus esophagogastrostomy performed by Japanese scholars (33), the incidence of reflux esophagitis after DTR was 12.5%. The results of all these studies are closer to ours.

In the LPG-TLR, after the tumor is removed with a cutter closure, the remnant stomach is cut into a tubular shape about 4 cm in width and 20 cm in length, and its length gradient and width make it less likely for food to reflux into the esophagus. The structure near the fundus of the cut remnant stomach can buffer and temporarily store the refluxed object, and the secretion of gastric acid is reduced because some of the stomach wall cells are removed. We believe that the above points make the LPG-TLR also have a superior anti-reflux effect than esophagogastrostomy. Because of its ease of procedure, it is a method that can be easily performed in most treatment centers.

### Postoperative nutritional status

Compared with total gastrectomy, proximal gastrectomy preserves the gastroduodenal channel. The pepsinogen secreted by the remnant stomach, together with the pancreatin and cholecystokinin secreted by the duodenum, promote the digestion and absorption of food, and thus the patient’s postoperative nutrition should theoretically be better than that of total gastrectomy. Also, the endocannabinoid secreted by the cells of the residual gastric wall helps vitamin B12 absorption in the distal ileum.

A study by Daisuke Ichikawa at Kyoto Medical University in Japan showed that hemoglobin and body weight levels were higher in the proximal gastrectomy group than in the total gastrectomy group 1-5 years after surgery (35). Similarly, another multicenter study (36), which included 254 patients, also showed that patients with double-tract reconstruction had better postoperative weight levels. The results of these studies are similar to our findings. However, there are fewer studies on proximal gastrectomy with LPG-TLR, which also indicates the greater significance of our study.

Hemoglobin and albumin in the LPG-TLR group decreased significantly at 6 months postoperatively compared with the preoperative levels, but they could recover to the preoperative level at 1 year postoperatively. In our study, there was a significant difference in weight between the two groups at 1 year postoperatively. Patients in the LPG-DTR group had significantly higher weight levels than those in the LPG-TLR group. And we will conduct a longer follow-up in future studies to verify the persistence of these differences.

### Quality of life after surgery

The postoperative QoL of patients is an important consideration in the choice of surgical modality. There are fewer studies on the quality of life of patients after proximal gastrectomy and equally few studies on the quality of life of LPG-TLR (37). In this study, we applied the Visick grade to comprehensively assess the postoperative QoL of patients, which is easy to assess and reflects the overall life status of patients. Visick I and Visick II are asymptomatic and have mild discomfort without additional intervention, respectively. In our present study, there was no significant difference in Visick grade in the overall comparison between the two groups, and the overall incidence of Visick I and II was 85.5% in the LPG-DTR group and 80.0% in the LPG-TLR group, indicating that most patients in both groups had no significant discomfort in the postoperative period. Overall, in terms of postoperative QoL, patients in both groups were predominantly graded II and below. Acceptable quality of life can be achieved with both digestive tract reconstruction modalities.

In conclusion, based on our findings and related studies, both LPG-DTR and LPG-TLR after proximal gastrectomy for AEG are safe and feasible. Both types of digestive tract reconstruction methods have comparable results in most of the perioperative indicators. The incidence of reflux esophagitis was not significantly higher in LPG-TLR patients than in the LPG-DTR patients. LPG-DTR had some advantages over LPG-TLR in terms of weight levels. Patients in both groups had a similar postoperative quality of life. Due to the limited sample volume of our study, the findings need to be further validated by larger samples and higher-quality prospective studies.

## Data availability statement

The original contributions presented in the study are included in the article/supplementary material. Further inquiries can be directed to the corresponding authors.

## Ethics statement

The studies involving human participants were reviewed and approved by the Ethics Committee of First Affiliated Hospital of Xiamen University. Written informed consent for participation was not required for this study in accordance with the national legislation and the institutional requirements.

## Author contributions

All authors listed have made a substantial, direct, and intellectual contribution to the work and approved it for publication.
